# News events and their relationship with US vape sales: an interrupted time series analysis

**DOI:** 10.1186/s12889-022-12858-x

**Published:** 2022-03-10

**Authors:** Kamila Janmohamed, Shinpei Nakamura-Sakai, Abdul-Nasah Soale, Laura Forastiere, Frederick L. Altice, Navin Kumar

**Affiliations:** 1 Yale College, New Haven, CT USA; 2 Department of Statistics, Yale University, New Haven, CT USA; 3 Department of Statistical Science, Fox School of Business, Temple University, Philadelphia, PA USA; 4 Department of Biostatistics, Yale School of Public Health, New Haven, CT USA; 5 Yale School of Medicine, Section of Infectious Diseases, 135 College Street, Suite 323, New Haven, 06510 CT USA

**Keywords:** News events, Vaping, E-cigarette, Harm reduction

## Abstract

**Objective:**

News coverage around vaping-related events may have furthered misconceptions regarding the relative harms of vapes. Such information may influence the decisions of individuals who smoke, around switching to vaping, potentially affecting the overall tobacco mortality burden. Thus, it is prudent to study how news events (e.g., 2019 vaping illness epidemic) are associated with vape sales in the United States, to possibly reduce the tobacco mortality burden.

**Methods:**

We used weekly retail sales data for e-cigarettes (30 December 2018 - 28 December 2019) from the US retail scanner data compiled by the Nielsen Company. We used an interrupted time series design with segmented regression analysis to determine immediate and longer-term impacts of individual news events (e.g. Trump administration’s planned ban on some flavored vaping products) on vape sales, controlling for pre-existing trends.

**Results:**

Unexpectedly, we noted a statistically significant positive relationship between vape sales and the CDC announcing an investigation into vaping-related illnesses (Change: 6.59%, Estimate: 0.066; 95% CI: 0.036, 0.092; P < 0.001). We also observed a similar positive association between vape sales and the CDC’s announcement on the link between Vitamin E acetate and EVALI (Change: 2.93%, Estimate: 0.029; 95% CI: 0.003, 0.055; P < 0.05). There was a steep decline in sales after these events.

**Conclusions:**

News events may be associated with US vape sales. Findings have implications for the management of risk perceptions around vaping to improve health outcomes of tobacco users. Information-based policy instruments can be applied to balance the effects of news events that may influence vape sales.

**Supplementary Information:**

The online version contains supplementary material available at (10.1186/s12889-022-12858-x).

## Introduction

E-cigarette use (vaping) is likely less injurious to health compared to combustible cigarettes, due to reduced production of toxic chemicals and carcinogens [1, 2]. Despite this evidence, many people who smoke in the US perceive e-cigarettes (vapes) to be at least as dangerous to health as combustible cigarettes [1, 3]. Such misconceptions may influence the decisions of people who smoke and are unable to quit, around switching to vaping [4] as a step toward smoking cessation. If an individual has been unsuccessful in attempts to quit smoking, switching to vaping may improve overall health outcomes [5, 6].

While youth vape use has declined since 2019, its prevalence remains high. As of 2020, 4.5% of US adults and 13.1% middle and high school students used e-cigarettes [7]. Sales from 2010-2016 show strong early growth followed by considerable slowing over time [8]. The US scientific consensus is that vape aerosol contains less toxicants than smoke from combustible tobacco cigarettes [9–11]. However, use of vapes results in dependence on nicotine, but with apparently less risk and severity than that of combustible tobacco cigarettes [9]. Among youths, vape use is associated with increased risk for cigarette initiation [12]. Among adults, a Cochrane review found that nicotine vapes probably do help people to stop smoking for at least six months, working better than nicotine replacement therapy and nicotine-free e-cigarettes [10]. Vaping related e-cigarette aerosol contains high concentrations of glycerol, -dodecalactone, and nicotine [13]. News events may have furthered misconceptions around the relative harms of vapes [14]. For example, during the outbreak of vaping-related lung injury (EVALI), media coverage resulted in a 130% increase in news articles warning against the dangers of vaping when the source of the epidemic was still unknown [15]. Later, it emerged that most cases were related to consumption of tetrahydrocannabinol (THC) added to vaping devices [14].

Vaping-related news events (e.g. CDC announcing an investigation into vaping-related illnesses on August 17 2019 following an outbreak in 14 states) can influence risk perceptions and normative perceptions around vaping [14, 16], especially around perceived risks of these products. We note that other factors can influence perceived risks, such as community, peers, word of mouth, advertising and promotion, and legislative regulation. Risk perceptions are associated with health-related behaviors and thus perceptions around vaping may influence use [17, 18]. Thus, news events may influence transitions from smoking to vaping, potentially affecting the overall tobacco mortality burden [19]. We also note that in some cases, vapes do not help adult smokers quit at rates higher than smokers who did not use these products[20].

We explored how various vaping-related news events (e.g. CDC announcing an investigation into vaping-related illnesses on August 17 2019 following an outbreak in 14 states, Trump administration plan to ban some flavored vaping products on September 11 2019, FDA’s warning to consumers against the use of vape products containing THC on October 4 2019, CDC’s announcement on the link between Vitamin E acetate and EVALI on November 8 2019) were associated with US vape sales. We aim to provide insight around improving health outcomes of people who smoke, amid increased risk perception around vaping [21, 22].

## Method

### Data

Weekly retail sales data for e-cigarettes (30 December 2018 - 28 December 2019) was obtained from US retail scanner data compiled by the Nielsen Company. Weekly sales data was denominated in US dollars (USD). This data represented weekly sales of e-cigarettes in Nielsen’s participating retailers, such as food, drug, mass stores, and convenience stores in a subset of 52 US states and other jurisdictions. Nielsen retail sales data does not include e-cigarette sales in non-participating retailers, vape stores and online e-cigarettes sales.

### Selection of events of interest

We assembled a list of vaping-related events based on a review of online news sites and peer-reviewed vaping research articles, and consultation with experts on vaping. First, two authors manually reviewed events to assess relevance to the study (Cohen’s Kappa > 90%), resulting in a long-list of events. Authors scored events based on three criteria: 1) relevance to vaping; 2) event discussed heavily in social media and other news media; 3) event discussed in academic articles. Only events meeting all criteria were selected. Examples of news sites are as follows: businessinsider.com/timeline-of-vape-related-illnesses-and-deaths-2019-9; https://www.beckershospitalreview.com/quality/how-vaping-turned-into-a-public-health-emergency-timeline-of-key-events.html; https://www.cdc.gov/tobacco/basicinformation/e-cigarettes/severe-lung-disease.html.

This process yielded five events as follows: 17 August 2019, CDC announced that they would be investigating cases of vaping-related illnesses; 11 September 2019, Trump administration considers ban on some flavored vaping products; 24 September 2019, Massachusetts bans vaping products; 4 October 2019, The FDA warned consumers not to use any THC-containing vapes; 8 November 2019, Vitamin E acetate responsible for EVALI (CDC announcement). We then identified key scholars in vaping through the number of articles (> 10) published regarding vaping. We contacted the identified researchers and asked them to assist. The researchers scored events based on the three criteria indicated earlier. Only events meeting all criteria were selected. The final four vaping-related news events were as follows: 1) CDC announcing an investigation into vaping-related illnesses on August 17 2019 following an outbreak in 14 states [23]; 2) Trump administration plan to ban some flavored vaping products on September 11 2019 [24]; 3) FDA’s warning to consumers against the use of vape products containing THC on October 4 2019 [25]; 4) CDC’s announcement on the link between Vitamin E acetate and EVALI on November 8 2019 [26].

### Statistical analysis

We used an interrupted time series design with segmented regression analysis to determine immediate and longer-term impacts of individual news events on vape sales, controlling for other time-dependent covariates (US-based hospitalizations from vaping, US weekly retail sales data for combustible cigarettes), as indicated below. We included US weekly retail sales data for combustible cigarettes as a covariate as cigarette sales trends are associated to vape sales trends [27, 28]. The dependent variable was log-transformed weekly retail sales data for e-cigarettes. Interrupted time series analysis can validate whether certain news events have an effect significantly greater than the underlying trend by collecting data at multiple instances overtime before and after news events [29]. Interrupted time series is the strongest quasi-experimental design to assess longitudinal effects of time-delimited treatments or interventions [30]. This design was appropriate as data was collected at multiple time points and we wanted to detect if an intervention (news events) had a significantly greater effect than another underlying trend [31]. The goal of interrupted time series analysis is to estimate the interaction terms between implementation of a news event and time. The regression coefficient on the interaction term is interpreted as the immediate impact on the level of the outcome (vape sales) [32]. Segmented regression analysis is a method for statistically modelling the interrupted time series data to draw more formal conclusions about the impact of an intervention or event on the measure of interest. In segmented regression analysis, each segment of the series is allowed to exhibit both a level and a trend. We examine the changes in trend that follows an event [33]. A change in trend after the event, constitutes an abrupt intervention (event) effect. A change in trend is defined by an increase or decrease in the slope of the segment after the event as compared with the segment preceding the event. A change in trend represents a gradual change in the value of the outcome during the segment. Individual models were not used for each event.

We first conducted a visual examination on the pattern of the time series by plotting them and generating auto-correlation and partial correlation plots. No seasonal patterns were identified. Auto-correlation was tested with the Durbin-Watson test. Nonstationarity was identified using the augmented Dickey-Fuller test and corrected through differencing. An autoregressive moving average (ARIMA) model of order 1 was fit against a white noise series generated from the stationarized data to determine optimal model parameters. Both models included binary variables for events (0=dates before the event, 1=dates after the event), time (1 was denoted for the first week and numbered sequentially after), and time since each event (1 was denoted for the first week after each event and numbered sequentially after). For example, after 10 weeks, the *time* variable would be 10. If there was an event that happened at the 10th week, the corresponding *time since each event* variable would be 1.

We used US-based hospitalizations from vaping and US weekly retail sales data for combustible cigarettes (denominated in USD) as control variables. We derived hospitalizations from vaping by summing the number of individuals hospitalized with lung injury associated with e-cigarette use or vaping in the US on a particular week, from CDC data [34]. Weekly retail sales data for combustible cigarettes was obtained from the US retail scanner data compiled by the Nielsen Company. This data represented weekly sales of cigarettes in Nielsen’s participating retailers. These control variables may address underlying time-varying factors possibly influencing vape sales. Including these factors may also control for pre-existing trends, essentially to avoid confusing a change due to these factors with a change due to the news events. By considering a broader picture of what may influence vape sales, we can better test the claims relation to the association between specific news events and vape sales. We calculated 95% confidence intervals for the association of each event with vape sales. We only reported results where the key predictor variable and its corresponding interaction term were significant at the P < 0.05 level. We also presented unadjusted results in Supplementary Table A. We predicted vape sales from 18 August 2019 to 28 December 2019, to better understand the trend of vape sales if the vaping-related events had not occurred, using data from 30 December 2018 to 17 August 2019 and the identical model above [ARIMA (1,1,0)]. We predicted vape sales to provide an understanding on how vape sales may have increased over time, given EVALI had not occurred. We used 18 August 2019 as the start of the prediction period as this was when the CDC announced their investigation of vape-related illnesses, which we took as the official start of EVALI and consequent shifts in vape sales. Analysis was conducted using R with the following packages: tseries, forecast and lmtest [35–37].

## Results

The mean weekly retail sales data for e-cigarettes (30 December 2018 - 28 December 2019) was $151,304,340 (SD=$15,813,160).

**Fig. 1:**
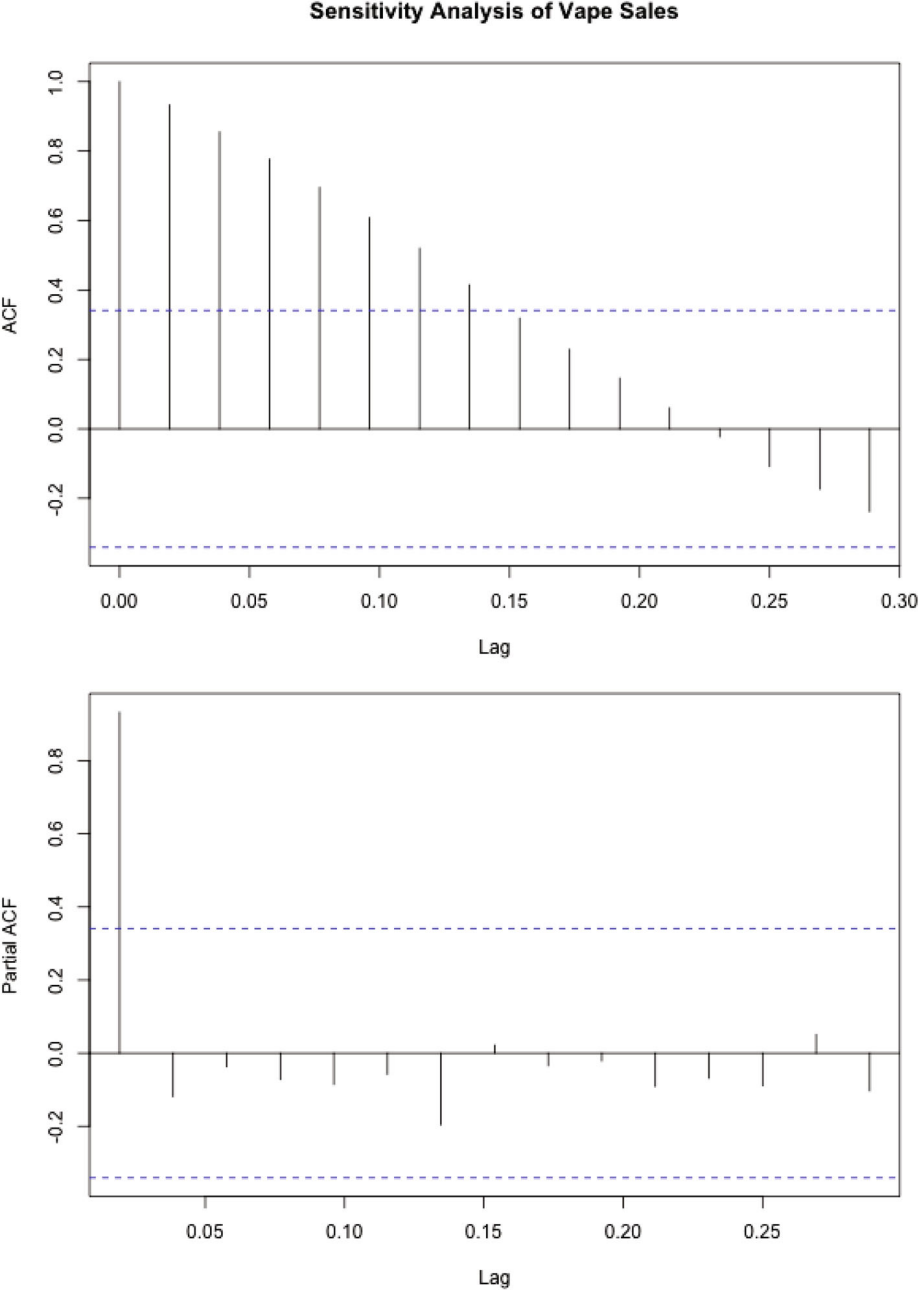
ACF and PACF plots for pre-EVALI e-cigarette sales data

Figure 1 illustrates the ACF and PACF plots for pre-EVALI e-cigarette sales data. The single statistically significant peak in the PACF plot indicates the suitability of a first-order ARIMA model. This is corroborated by the Durbin-Watson test (P < 0.001), which suggests that true auto-correlation in the model is greater than 0. Furthermore, the Augmented Dickey-Fuller test (See Supplementary Table C) indicates that the series is stationary only for a model with drift, trend and 0 lags.

**Fig. 2:**
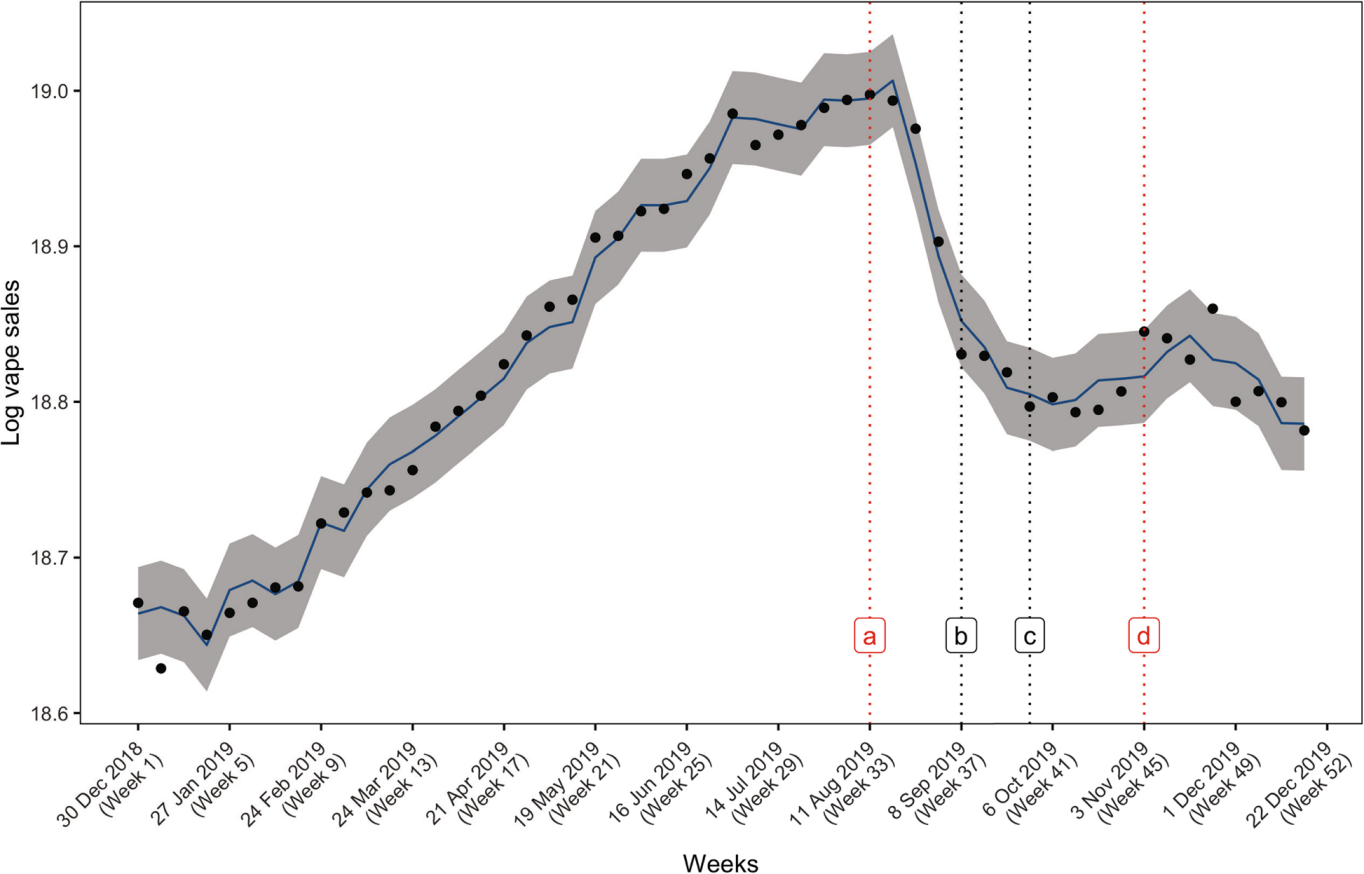
Vape sales scatterplot and trends with a timeline of vaping-related news events. (a) August 17 2019: CDC announces investigation into vaping-related illnesses (b) September 11 2019: Trump administration considers ban on some flavored vaping products (c) October 4 2019: FDA warns against using vape products containing THC (d) November 8 2019: CDC announces relationship between Vitamin E acetate and lung injury outbreak Note. Association between vape sales and exposure to news events. The solid line indicated the smoothed weekly vape sales with the corresponding confidence interval (in grey) and was obtained by fitting the date of the vape sales to the amount of vape sales using an interrupted time series design with segmented regression analysis. Red boxes denoted news events that had statistically significant association (p < 0.05) with shifts in vape sales

Figure 2 illustrates vape sales over time (30 December 2018 - 28 December 2019). Vape sales were originally increasing. There was then a steep decline. Sales rose after FDA’s warning to consumers against the use of vape products containing THC (October 4 2019), with a continued increase after CDC’s announcement on the link between Vitamin E acetate and EVALI (November 8 2019). There was a subsequent decline shortly after CDC’s announcement on the link between Vitamin E acetate and EVALI.

Table 1 reports the estimates of the interrupted time series design with segmented regression analysis across various news events (See Supplement for full results). We noted a statistically significant positive relationship between vape sales and the CDC announcing an investigation into vaping-related illnesses (Change: 6.59%, Estimate: 0.066; 95% CI: 0.036, 0.092; P < 0.001). We observed a similar positive association between vape sales and the CDC’s announcement on the link between Vitamin E acetate and EVALI (Change: 2.93%, Estimate: 0.029; 95% CI: 0.003, 0.055; P < 0.05). All events were associated with statistically significant slope changes. The Trump administration planning to ban some flavored vaping products (0.038, P < 0.001) and the FDA warning consumers against the use of vape products containing THC (0.029, P < 0.001) were associated with a slope increase. The CDC announcing an investigation into vaping-related illnesses (-0.064, P < 0.001) and the CDC announcing the link between Vitamin E acetate and EVALI (-0.022, P < 0.001) were associated with a slope decline. We focus our discussion of these results on changes in level of vape sales, given our interest in changes occurring directly after specific events. Unadjusted estimates (Supplementary Table A) also show a statistically significant positive relationship between vape sales and the CDC announcing an investigation into vaping-related illnesses (Change: 6.59%, Estimate: 0.066; 95% CI: 0.033, 0.099; P < 0.001), with a slope change of -0.067 (P < 0.001). Full results in Supplementary Table B indicated that all control variables, except EVALI Hospitalizations, were statistically significant.

**Table 1:** Estimates of the interrupted time series design with segmented regression analysis across various vaping-related events

Event	Estimate(95% CI)	p	Interaction(95% CI)	p interaction
CDC announcing an investigation into vaping-related illnesses	0.066(0.036, 0.092)	**p < 0.001**	-0.064(-0.075, -0.052)	**p < 0.001**
Trump administration plan to ban some flavored vaping products	0.014(-0.018, 0.045)	0.398	0.038 (0.020, 0.056)	p < 0.001
FDA warns consumers against the use of vape products containing THC	-0.006(-0.034, 0.021)	0.651	0.029 (0.012, 0.046)	p < 0.001
CDC announces link between Vitamin E acetate and EVALI	0.029(0.003, 0.055)	**0.029**	-0.022(-0.033, -0.010)	**p < 0.001**

Bold *p*-values indicate statistical significance (P < 0.05) for both the news event (key dependent variable) and the corresponding interaction term (P < 0.05)

**Fig. 3:**
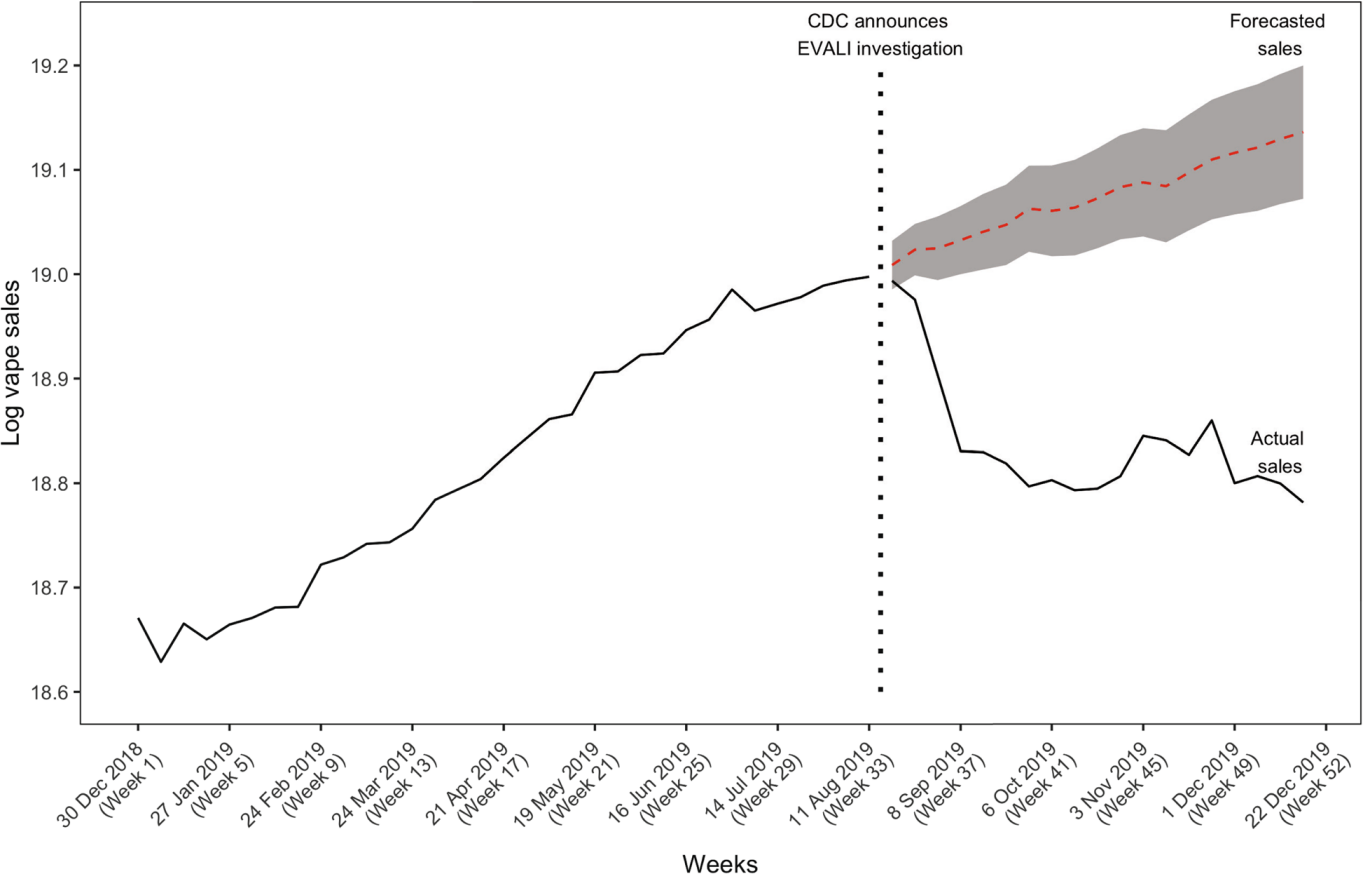
Vape sales forecast with ARIMA (1,1,0) assuming EVALI events had not occurred. Note. Red dotted line and corresponding confidence interval (in grey) represented forecast with identical ARIMA (1,1,0) model as Figure 1. The black lines indicated actual weekly vape sales

Figure 3 details predicted vape sales assuming the vaping-related events had not occurred. Results indicated a continued increase in forecasted sales, suggesting that EVALI resulted in a 21% decrease in total sales worth $767,099,216.

## Discussion

We found associations between certain news events and changes in US vape sales. The strength of our work is the use of robust statistical methods to explore how news events are associated with US vape sales. Such outcome measurement is central to understanding how news events may shift vape sales. Public health authorities can also conduct interventions to balance the rhetoric of news events, developing messaging based on past qualitative work [38, 39]. Interventions that ask respondents to judge information accuracy around vaping [40, 41], may nudge individuals toward accurate information regarding vaping during news events which possibly distort vaping perceptions. Future research can detail how some news events have a greater effect on vape sales compared to others, and address how best to intervene around disproportionate responses to vaping news events.

A key limitation is that we cannot say with certainty that news events caused a shift in vape sales or whether there were other underlying factors. It is possible that the change in intercept results could be spurious artefacts of the way the model has been constructed. We provide correlational evidence, but cannot make causal claims. We also limited in our ability to adjust for other possible confounders e.g. sales of nicotine replacement produces or cannabis-related sales. We were unable to measure other factors that may have a role on vape sales, such as community and peer influence, word of mouth, advertising and promotion, and legislative regulation. We were unable to measure the effect of legislation or regulation, such as the FDA ban on flavored vapes [42], on changes in vape sales. We were not able to account for the connotation of media messages using techniques such as sentiment analysis [43]. The data was also restricted to a single year, possibly hindering ability to understand impacts of events in relation to longer-term trends. We note the measurement error arising from the exclusion of Nielsen retail e-cigarette sales data of non-participating retailers and online vape sales may introduce bias which may have varied over the study time period. Consideration should be given to the potential distorting effect of the USD denomination of vape sales data – whether price rises over the one-year period may account, in part, for the decreases detected.

We indicated that news events may be associated with changes in US vape sales. Findings have implications for the management of risk perceptions around vaping to improve health outcomes of tobacco users. Information-based policy instruments can be applied to balance the negative effects of news events that may affect vape sales.

## Supplementary Information

**Additional file 1** Supplementary Table A–C.

## Data Availability

The datasets used and analyzed during the current study available from the corresponding author on reasonable request.

## References

[1] McNeill A, Brose LS, Calder R, Bauld L, Robson D. Evidence review of e-cigarettes and heated tobacco products 2018. A report commissioned by Public Health England. Lond: Public Health England. 2018; 6.

[2] Zettler PJ, Hemmerich N, Berman ML (2018). Closing the regulatory gap for synthetic nicotine products. Boston College law review. Boston College. Law School.

[3] Nyman AL, Huang J, Weaver SR, Eriksen MP (2019). Perceived comparative harm of cigarettes and electronic nicotine delivery systems. JAMA Netw Open.

[4] Tattan-Birch H, Brown J, Shahab L, Jackson SE. Association of the us outbreak of vaping-associated lung injury with perceived harm of e-cigarettes compared with cigarettes. JAMA Netw Open. 2020; 3(6). 10.1001/jamanetworkopen.2020.6981. 10.1001/jamanetworkopen.2020.6981 PMC7296387 32539148

[5] Polosa R, Morjaria JB, Caponnetto P, Prosperini U, Russo C, Pennisi A, Bruno CM. Evidence for harm reduction in copd smokers who switch to electronic cigarettes. Respir Res. 2016; 17(1). 10.1186/s12931-016-0481-x. 10.1186/s12931-016-0481-x PMC5162097 27986085

[6] Polosa R, Cibella F, Caponnetto P, Maglia M, Prosperini U, Russo C, Tashkin D. Health impact of e-cigarettes: a prospective 3.5-year study of regular daily users who have never smoked. Sci Rep. 2017; 7(1). 10.1038/s41598-017-14043-2. 10.1038/s41598-017-14043-2 PMC5693960 29150612

[7] Park-Lee E, Ren C, Sawdey MD, Gentzke AS, Cornelius M, Jamal A, Cullen KA (2021). Notes from the field: E-cigarette use among middle and high school students—national youth tobacco survey, united states, 2021. Morb Mortal Wkly Rep.

[8] Cantrell J, Huang J, Greenberg M, Willett J, Hair E, Vallone D (2020). History and current trends in the electronic cigarette retail marketplace in the united states: 2010–2016. Nicotine Tob Res.

[9] Daynard R. Public health consequences of e-cigarettes: a consensus study report of the National Academies of Sciences, Engineering, and Medicine.2018. p. 379–381.

[10] Hartmann-Boyce J, McRobbie H, Butler AR, Lindson N, Bullen C, Begh R, Theodoulou A, et al.Electronic cigarettes for smoking cessation. Cochrane Database Syst Rev. 2021; 9. 10.1002/14651858.CD010216.pub4 PMC8094228 33052602

[11] Drope J, Cahn Z, Kennedy R, Liber AC, Stoklosa M, Henson R, Douglas CE, Drope J (2017). Key issues surrounding the health impacts of electronic nicotine delivery systems (ends) and other sources of nicotine. CA Cancer J Clin.

[12] Berry KM, Fetterman JL, Benjamin EJ, Bhatnagar A, Barrington-Trimis JL, Leventhal AM, Stokes A (2019). Association of electronic cigarette use with subsequent initiation of tobacco cigarettes in us youths. JAMA Netw Open.

[13] Su W-C, Lin Y-H, Wong S-W, Chen JY, Lee J, Buu A. Estimation of the dose of electronic cigarette chemicals deposited in human airways through passive vaping. J Expo Sci Environ Epidemiol; 31(6):1008–16. 10.1038/s41370-021-00362-0 PMC8595527 34239037

[14] Hall W, Gartner C, Bonevski B (2021). Lessons from the public health responses to the us outbreak of vaping-related lung injury. Addiction.

[15] Leas EC, Nobles AL, Caputi TL, Dredze M, Zhu S-H, Cohen JE, Ayers JW. News coverage of the e-cigarette, or vaping, product use associated lung injury (evali) outbreak and internet searches for vaping cessation. Tob Control. 2020. 10.1136/tobaccocontrol-2020-055755. 10.1136/tobaccocontrol-2020-055755 PMC8041908 33051278

[16] Dave D, Dench D, Kenkel D, Mathios A, Wang H. News that takes your breath away: Risk perceptions during an outbreak of vaping-related lung injuries. 2020. 10.3386/w26977. 10.1007/s11166-020-09329-2 PMC8425473 34504389

[17] Minton EA, Gardiner P. The missing role of moral values in anti-vaping messaging. J Consum Aff; 55(3):1040–61.

[18] Pepper JK, Squiers LB, Peinado SC, Bann CM, Dolina SD, Lynch MM, Nonnemaker JM, McCormack LA (2019). Impact of messages about scientific uncertainty on risk perceptions and intentions to use electronic vaping products. Addict Behav.

[19] Leas EC, Nobles AL, Caputi TL, Dredze M, Zhu S-H, Cohen JE, Ayers JW (2021). News coverage of the e-cigarette, or vaping, product use associated lung injury (evali) outbreak and internet searches for vaping cessation. Tob Control.

[20] Weaver SR, Huang J, Pechacek TF, Heath JW, Ashley DL, Eriksen MP (2018). Are electronic nicotine delivery systems helping cigarette smokers quit? evidence from a prospective cohort study of us adult smokers, 2015–2016. PLoS ONE.

[21] Dave D, Dench D, Kenkel D, Mathios A, Wang H (2020). News that takes your breath away: risk perceptions during an outbreak of vaping-related lung injuries. J Risk Uncertain.

[22] Kreslake JM, Diaz MC, Shinaba M, Vallone DM, Hair EC (2022). Youth and young adult risk perceptions and behaviours in response to an outbreak of e-cigarette/vaping-associated lung injury (EVALI) in the USA. Tob Control.

[23] Centers for Disease Control and Prevention. CDC, states investigating severe pulmonary disease among people who use e-cigarettes. 2019. Accessed 30 June 2020. https://www.cdc.gov/media/releases/2019/s0817-pulmonary-disease-ecigarettes.html.

[24] The White House. Remarks by President Trump in Meeting on E-Cigarettes. 2019. Accessed 30 June 2020. https://www.whitehouse.gov/briefings-statements/remarks-president-trump-meeting-e-cigarettes/.

[25] Berke J. The FDA just leveled a stark warning against using any vapes containing THC amid an outbreak of lung disease. 2019. Accessed 30 June 2020. https://www.businessinsider.com/fda-warns-consumers-not-to-use-marijuana-vapes-amid-lung-disease-crisis-2019-10.

[26] Grady D. Vaping Illnesses Are Linked to Vitamin E Acetate, C.D.C. Says. 2019. Accessed 30 June 2020. https://www.nytimes.com/2019/11/08/health/vaping-illness-cdc.html.

[27] Yao T, Sung H-Y, Huang J, Chu L, Helen GS, Max W (2020). The impact of e-cigarette and cigarette prices on e-cigarette and cigarette sales in california. Prev Med Rep.

[28] Marynak KL, Gammon DG, King BA, Loomis BR, Fulmer EB, Wang TW, Rogers T (2017). National and state trends in sales of cigarettes and e-cigarettes, us, 2011–2015. Am J Prev Med.

[29] Ramsay CR, Matowe L, Grilli R, Grimshaw JM, Thomas RE (2003). Interrupted time series designs in health technology assessment: lessons from two systematic reviews of behavior change strategies. Int J Technol Assess Health Care.

[30] Peng RD, Dominici F, Louis TA. Journal of the Royal Statistical Society: Series A (Statistics in Society). 2006; 169(2):179–203.

[31] Kontopantelis E, Doran T, Springate DA, Buchan I, Reeves D (2015). Regression based quasi-experimental approach when randomisation is not an option: interrupted time series analysis. BMJ.

[32] Penfold RB, Zhang F (2013). Use of interrupted time series analysis in evaluating health care quality improvements. Acad Pediatr.

[33] Wagner AK, Soumerai SB, Zhang F, Ross-Degnan D (2002). Segmented regression analysis of interrupted time series studies in medication use research. J Clin Pharm Ther.

[34] Centers for Disease Control and Prevention and others. Outbreak of lung injury associated with e-cigarette use, or vaping. 2019. Accessed 30 June 2020. https://www.cdc.gov/media/releases/2019/s0817-pulmonary-disease-ecigarettes.html.

[35] Trapletti A, Hornik K. Tseries: Time Series Analysis and Computational Finance. 2019. R package version 0.10-47. https://CRAN.R-project.org/package=tseries. Accessed 5 Jan 2020.

[36] Hyndman R, Athanasopoulos G, Bergmeir C, Caceres G, Chhay L, O’Hara-Wild M, Petropoulos F, Razbash S, Wang E, Yasmeen F. forecast: Forecasting Functions for Time Series and Linear Models. 2020. R package version 8.12. http://pkg.robjhyndman.com/forecast. Accessed 5 Jan 2020.

[37] Hothorn T, Zeileis A, Farebrother RW, Cummins C, Millo G, Mitchell D. Lmtest: Testing Linear Regression Models. 2019. R package version 0.9-37. https://cran.r-project.org/web/packages/lmtest. Accessed 5 Jan 2020.

[38] Popova L, Fairman RT, Akani B, Dixon K, Weaver SR (2021). “don’t do vape, bro!” a qualitative study of youth’s and parents’ reactions to e-cigarette prevention advertisements. Addict Behav.

[39] Fairman RT, Weaver SR, Akani BC, Dixon K, Popova L (2021). “ you have to vape to make it through”: E-cigarette outcome expectancies among youth and parents. Am J Health Behav.

[40] Pennycook G, Rand DG (2019). Fighting misinformation on social media using crowdsourced judgments of news source quality. Proc Natl Acad Sci.

[41] Barnett PA, Hoskins CE, Alhoti JA, Carpenter LJ (2009). Reducing public misinformation about organ donation: An experimental intervention. J Soc Distress Homeless.

[42] Tanne JH. FDA bans most flavoured e-cigarettes as lung injury epidemic slows. BMJ. 2020;368:m12. 10.1136/bmj.m12. 10.1136/bmj.m12 31900244

[43] Pang B, Lee L (2008). Opinion mining and sentiment analysis. Foundations TrendsⓇ in information retrieval.

