# Supplementary material for: News events and their relationship with US vape sales: an interrupted time series analysis
Source: BMC Public Health. 2022 Mar 10;22:479. doi: 10.1186/s12889-022-12858-x (PMC8915465; doi:10.1186/s12889-022-12858-x)
Supplement: Supplementary file 1 — Additional file 1 Supplementary Table A–C. [file 12889_2022_12858_MOESM1_ESM.pdf]

Supplementary Table A: Unadjusted model of the interrupted time series design with segmented regression analysis across various vaping-related events

**Variable**	**Estimate (95% CI)**	**p**	**Interaction (95% CI)**	**p interaction**
AR(1)	-0.550 (-0.809, -0.291)	p<0.001	N/A	N/A
Weeks	0.011 (0.007, 0.014)	p<0.001	N/A	N/A
CDC announcing an investigation into vaping-related illnesses	0.066 (0.033, 0.099)	**p<0.001**	-0.067 (-0.081, -0.054)	**p<0.001**
Trump administration plan to ban some flavored vaping products	0.007 (-0.029, 0.044)	0.692	0.043 (0.022, 0.064)	p<0.001
FDA warns consumers against the use of vape products containing THC	-0.016 (-0.048, 0.016)	0.327	0.024 (0.004, 0.044)	0.018
CDC announces link between Vitamin E acetate and EVALI	0.016 (-0.014, 0.045)	0.309	-0.020 (-0.033, -0.006)	p<0.001

Bold p-values indicate statistical significance (P<0.05) for both the news event (key dependent variable) and the corresponding interaction term (P<0.05).

Supplementary Table B: Full model of the interrupted time series design with segmented regression analysis across various vaping-related events

**Variable**	**Estimate (95% CI)**	**p**	**Interaction (95% CI)**	**p interaction**
AR(1)	-0.576 (-0.836, -0.316)	p<0.001	N/A	N/A
Weeks	0.009 (0.006, 0.013)	p<0.001	N/A	N/A
EVALI hospitalizations	-0.002 (-0.007, 0.003)	0.527	N/A	N/A
Log weekly cigarette sales	0.546 (0.296, 0.796)	p<0.001	N/A	N/A
CDC announcing an investigation into vaping-related illnesses	0.066 (0.036, 0.092)	**p<0.001**	-0.064 (-0.075, -0.052)	**p<0.001**
Trump administration plan to ban some flavored vaping products	0.014 (-0.018, 0.045)	0.398	0.038 (0.020, 0.056)	p<0.001
FDA warns consumers against the use of vape products containing THC	-0.006 (-0.034, 0.021)	0.651	0.029 (0.012, 0.046)	p<0.001
CDC announces link between Vitamin E acetate and EVALI	0.029 (0.003, 0.055)	**0.029**	-0.022 (-0.033, -0.010)	**p<0.001**

Bold p-values indicate statistical significance (P<0.05) for both the news event (key dependent variable) and the corresponding interaction term (P<0.05).

Supplementary Table C: Augmented Dickey Fuller Test Results

Lag	ADF	p value
Type 1: No drift no trend		
0	3.72	0.99
1	5.93	0.99
2	2.94	0.99
3	2.90	0.99
Type 2: With drift no trend		
0	0.131	0.961
1	-0.165	0.929
2	-0.352	0.904
3	-0.467	0.870
Type 3: With drift and trend		
0	-3.88	0.0274
1	-1.67	0.6927
2	-2.00	0.5580
3	-1.52	0.7537
